# Integration of an anti-tumor drug into nanocrystalline assemblies for sustained drug release†
†Electronic supplementary information (ESI) available. See DOI: 10.1039/c4sc03392b



**DOI:** 10.1039/c4sc03392b

**Published:** 2015-01-06

**Authors:** Xiangrui Yang, Shichao Wu, Yang Li, Yu Huang, Jinyan Lin, Di Chang, Shefang Ye, Liya Xie, Yuan Jiang, Zhenqing Hou

**Affiliations:** a Institute of Soft Matter and Biomimetics , College of Materials , Xiamen University , Xiamen 361005 , China . Email: houzhenqing@xmu.edu.cn ; Email: yuan.jiang@xmu.edu.cn ; Fax: +86-592-2183058; b Department of Chemistry , College of Chemistry & Chemical Engineering , Xiamen University , Xiamen 361005 , China; c The First Affiliated Hospital of Xiamen University , Xiamen University , Xiamen 361003 , China

## Abstract

Delicate mesoscopic architectures, bearing complex forms with multiple hierarchy levels, lead to significant functions in biogenic minerals. Herein, a bio-inspired approach was developed to fabricate comet-shaped assemblies of an anti-tumor drug – 10-hydroxycamptothecin (HCPT). The anti-solvent co-precipitation of HCPT and the excipient – PEG-*b*-PLGA – within the emulsifier leads to the immediate nucleation of comet bundles, followed by a secondary nucleation to generate the comet head, which is an assembly of nanofibers aligned almost in parallel. The continuous manufacturing furnishes drug–excipient hybrid particles with high drug-loading and a sustained drug release profile. This simple and efficient bio-inspired approach led to a promising sustained local drug delivery system, and could be extended to the fabrication of other functional organic materials bearing mesoscopic structural units.

## Abstract 2

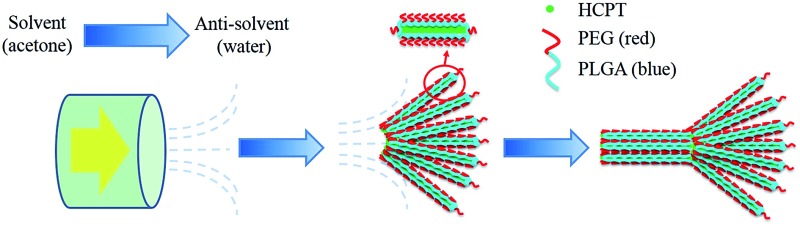
A bio-inspired approach was used to integrate an anti-tumor drug into nanocrystalline assemblies for sustained drug release.

## Introduction

The delicate hierarchical architectures of biogenic materials across meso- and macroscales suggest the existence of an alternative non-classical crystallization route.^1^ Instead of passing through the straightforward classical crystallization pathway, where crystals grow *via* atomic/molecular attachments, the precursor formation and transformation into crystalline species proceed predominantly *via* non-classical crystallization routes. Non-classical crystallization, usually a multistage process, not only dominates in biomineralization,^1,2^ but was overlooked in normal crystallization procedures for a long time. The precursors could have multiple forms. They could be non-crystalline species including clusters,^3–6^ amorphous nanoparticles,^7–9^ or liquid-like droplets.^10–13^ In addition, they can be crystalline in form including nanocrystals^14–17^ or mesocrystals.^18,19^ The transiently existing precursors pass through the coupled crystallization-assembly process before reaching the thermodynamically stable crystalline form.^19,20^ For instance, the precipitation of alanine within the proper pH range could pass through the porous mesocrystalline state, as detected using the small-angle neutron scattering technique.^19^ Soft ingredients can be actively involved *via* chemical modification with supramolecular side chains or the physical adsorption of surfactants or polyelectrolytes. Both possibilities enrich the multistage crystallization pathways and the structural outcomes by stabilizing the nanospecies and guiding the assembly behavior of the target compounds.^21–24^ For instance, supramolecular porphyrins can assemble into nanofibers, which continue to assemble spatially into microplatelets under the guidance of a block copolymer additive – Pluronic F127.^22^ In comparison, supramolecular porphyrin would grow into bulk crystals without the amphiphilic additive.^24^ The fabrication of functional materials *via* multistage crystallization pathways is promising to achieve advanced materials bearing rich mesoscopic forms and functions closely related to their mesoscopic structural forms.

For instance, multistage crystallization could have a profound and lasting impact on nanodrug manufacturing.^25^ Nevertheless, there exist two main hurdles in fabricating nanodrugs: the involvement of high amounts of surfactants as stabilizers and the long-term instability of nanodrugs. Hence, the manipulation of nanodrugs with high drug loadings and a high stability is of great importance. The organization of nanospecies into assemblies would be a promising option, where a small amount of stabilizers including surfactants,^22,26^ polyelectrolytes,^27–30^ and (block) copolymers^22,31^ could provide the colloidal forces to stabilize the assemblies and to prevent adjacent nanocrystals from merging into continuous architectures. This approach has been successful in fabricating a series of functional materials including metal oxides,^32^ organics,^29,31^ and metal carbonates/phosphates^23,33^ into assemblies of nanocrystals. Significantly, the assembly could be well-guided using external fields, which could induce the translational and orientational orders towards assembly at the initial assembly stage.^34,35^ Unlike their nanocrystalline counterparts, assemblies of nanocrystalline drugs, usually micrometers in size, are advantageous for the separation with current tools and could be a clever option to escape the nanotoxicity problems.

Here, we report the fabrication of assemblies of nanocrystalline 10-hydroxycamptothecin (HCPT) – a promising broad-spectrum anti-tumor agent with remarkable success in early clinical trials – *via* a multistage crystallization process. The anti-solvent co-precipitation of HCPT and the excipient PEG-*b*-PLGA^36–38^ (monomethoxy polyethylene glycol)-*block*-poly(lactide-*co*-glycolide) (PEG: 10 wt%, 5000 Da; PLGA: 90 wt%, 28 000 Da) leads to the nucleation of hybrid nanofibers with nanocrystalline HCPT as the core wrapped with different amounts of PEG-*b*-PLGA as the steric stabilizer, depending on the initial ratios of active pharmaceutical ingredient (API)/excipient. Simultaneously, the flow within the channels of the emulsifier induces the spatial alignment of nanofibers into assemblies of several micrometers in size. Delightfully, the continuous manufacturing of assemblies of nanocrystalline HCPT in this one-step process achieves HCPT–PEG-*b*-PLGA hybrid assemblies with an enhanced effect of sustained drug release for tumor treatments compared to both their nanocrystalline and bulk counterparts.

## Results and discussion

A key feature of these experiments is the performance of the anti-solvent crystallization within a SPG membrane emulsification kit (SPG Technology Co. Ltd., Miyazaki, Japan). Both HCPT and PEG-*b*-PLGA were first dissolved in acetone and stored in the dispersion phase. Afterwards, a high-pressure N_2_ flow pushed the dispersion solution through the SPG membrane to enter the bulk aqueous phase, which is a poor solvent for both HCPT and PEG-*b*-PLGA. Due to the good miscibility of water and acetone and the existence of a strong fluid force, the concentration gradient of the solvent mixture was readily achieved in the exit area of each channel. Hence, the nucleation of HCPT nanofibers and the accompanying co-precipitation of PEG-*b*-PLGA onto the growing HCPT nanofibers presumably occurred in the exit area where the steep increase of the water content immediately increased the local supersaturation of both components to generate co-precipitation. The dynamic nucleation and precipitation of the nanospecies within the channel, plus the active occlusion of the soft ingredient PEG-*b*-PLGA and the experimental conditions used, led to assemblies of the nanocrystalline HCPT with tunable morphological character and componential distribution.

**Scheme 1:**
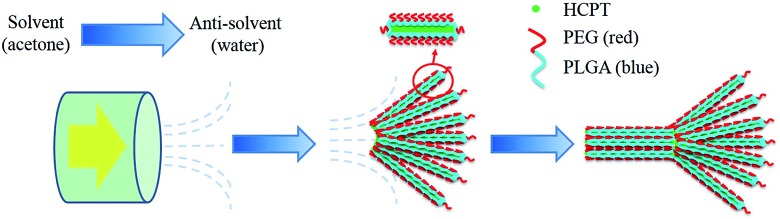
Illustration of the multistage crystallization of the comet-shaped particles within the emulsifier. The color gradient in the blue arrow indicates the increase of HCPT supersaturation, which increases with brighter color.

A multistage crystallization pathway was proposed for the formation of the comet-shaped particles (Scheme 1). Although the co-precipitation of the PEG-*b*-PLGA molecules, due to their insolubility in water, coats a thin layer of the block copolymer onto the growing HCPT nanofibers, its unsymmetric distribution on the nanofibers still allows for the further growth of hybrid nanofibers on both ends, where the polymer density is relatively low. Considering that the crystallization depletes the amount of surrounding HCPT molecules, further growth of the nanofibers prefers to occur in the rear areas (the head of each bundle) to form the comet head, composed of almost parallel-aligned nanofibers, due to the high local supersaturation. The morphological details may vary according to the experimental conditions, which will be discussed together with the microscopic images. The assemblies are permanently preserved because of the strong depletion force between adjacent nanofibers in analogy to the porphyrin microplatelets^22^ and BaSO_4_ fiber bundles.^39^ The existence of an external fluid force is crucial for the morphological control in the multistage crystallization process. In comparison, the anti-solvent co-precipitation of HCPT and PEG-*b*-PLGA in the bulk phase under stagnant conditions led to spherulitic microspheres (see ESI material†). The introduction of sonication produced a dispersion containing well-dispersed hybrid nanorods, which slowly aggregated after the sonication force was removed (see ESI material†). Hence, the dynamic solvent mixing at the interface of membrane and aqueous phase is responsible for the comet-like shape.

Nevertheless, there could be another scenario for the multistage crystallization, which is based on the formation of the comet head within the channel followed by the crystallization of the comet tail. A simple experiment was designed to exclude this possibility. Instead of using the HCPT–PEG-*b*-PLGA acetone mother liquor as the dispersion phase, we pumped a freshly prepared HCPT nanorod acetone–water dispersion through a filter membrane with a similar diameter and checked the morphology of the collected particles. All particles obtained were dumbbell-shaped particles with a spherulitic tail on each end, suggesting that the crystallization occurred on both ends of the nanorods in the exit area of the channel followed by overgrowth of the nanofibers in the tails (see ESI material†). This result unambiguously excludes the second pathway because the nucleation within the channel will still achieve comet-shaped particles with the prolonged comet head after passing through the channel.

The existence of PEG-*b*-PLGA possibly may not change either the crystal form or the morphology of HCPT because a similar procedure without PEG-*b*-PLGA also passes through the formation of nanofibers before recrystallization into bulk crystals. In each hybrid nanofiber, the PLGA chains prefer attaching to the inner HCPT nanofibers due to their hydrophobicity, while the short PEG segments (5000 Da) are exposed to the water owing to their relative hydrophilicity and serve as the temporary steric stabilizer to stabilize the hybrid nanofibers from too fast and uncontrolled aggregation. This temporary stabilization was already utilized for inorganic nanocrystals using double hydrophilic block copolymers to allow a controlled superstructure formation.^40^ For these polymers, PEG blocks with a length of 3000–5000 g mol^–1^ were applied and striking examples of nanocrystal superstructures exist including BaSO_4_ fiber bundles.^39^

**Fig. 1:**
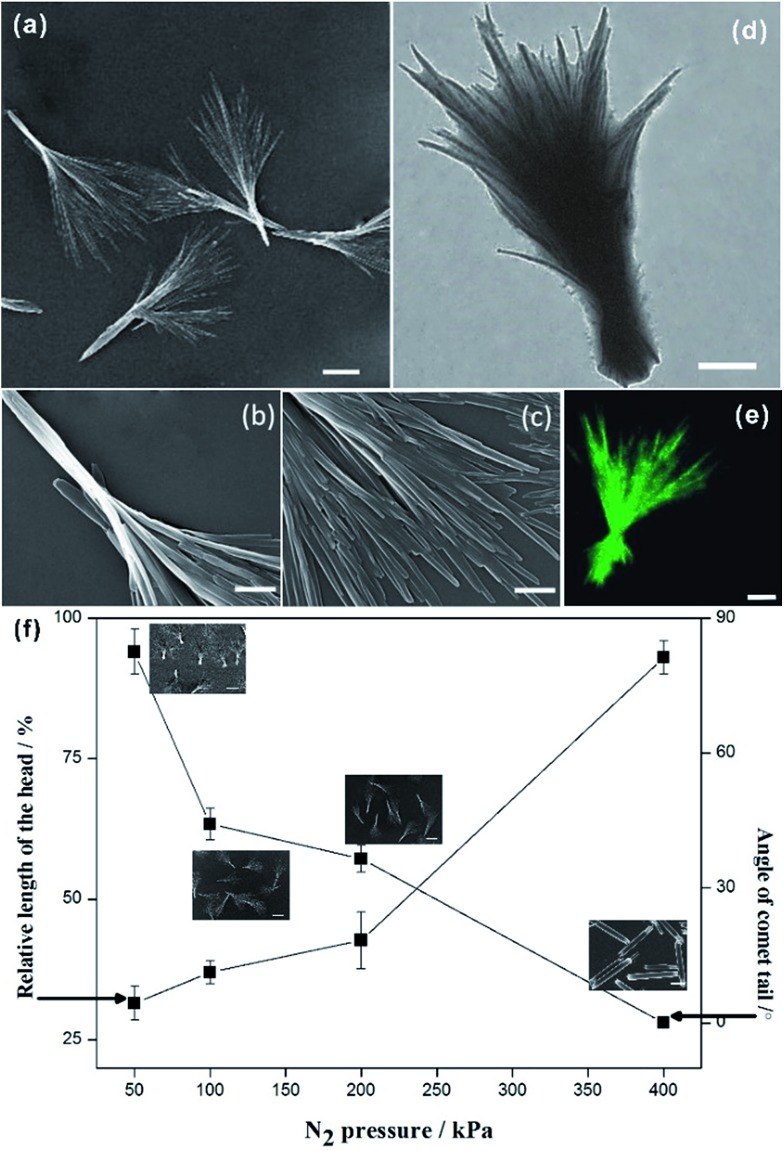
Images a–e illustrate the morphology of the comet-shaped particles. The SEM image a is an overview of the comet-shaped particles. The SEM images b and c show the structural details of one particle. Images d and e are TEM and CLSM images of a comet-shaped particle. The scale bars in a–e represent 5 μm, 700 nm, 500 nm, 2 μm, and 2 μm. Image f shows a graph with the corresponding SEM images, indicating the relationship between the N_2_ pressure and the tail angle (the right axis) as well as the relative length (head/tail) of the comet head (the left axis). The scale bars of the SEM images in f represent 5 μm.

The structural details of the comet-shaped particles were effectively recorded using optical and electron microscopes. SEM images show that the particles are 10–15 μm in length with a width of the comet head of about 400 nm in size. In particular, the comets' tail consists of a bundle of nanofibers aligned in a radiated fashion from the main body of the particle. Interestingly, the shape of the tail is dependent on the flow rate controlled by the N_2_ pressure. Increasing the N_2_ pressure causes a decreased angle of the tail and an increased length of the comet head, as shown in Fig. 1f. The angle of the tail and the length of the head reflect the flow rate-dependent crystallization of the growing HCPT nanofibers in the bulk aqueous phase. Hence, the nanofibers in the tail certainly spend a limited time in the supersaturated regime to grow fully, and comet-like particles with a narrow tail can be obtained. Additionally, the strong flow rate during the crystallization also assists in obtaining relatively narrow tails. The limited crystallization time under a strong N_2_ pressure keeps the HCPT supersaturation in the acetone–water mixture relatively high. Thus, a long comet head is achieved. In an extreme case, the tail area almost disappears when the N_2_ pressure is as high as 400 kPa (see ESI material†). Interestingly, the nanofibers in the comet head are partially merged into single crystalline architectures because of all the time for the growth of the comet head and the limited protection from PEG-*b*-PLGA, in analogy to the supramolecular porphyrin growth lacking enough amphiphilic additives.^24^ The single crystalline microfibers, owing to their limited surface area, showed a decrease in the drug release effect compared with their comet-shaped counterparts (see single crystalline properties of microfibers in the polarized optical microscopic images in the ESI material†). So, such particles are not assemblies of nanocrystals and, hence, have been excluded from the drug delivery experiments.

The co-precipitation of HCPT and PEG-*b*-PLGA produces hybrid particles with a variable drug loading from 11.9 to 48.3%. To obtain the highest entrapment efficiency and drug-loading content, an orthogonal L9 (3^3^) test was designed to optimize the formulation conditions (see ESI material†). The highest values of the entrapment efficiency and the drug loading are 96.6% and 48.3%, respectively, under the following experimental conditions: HCPT/PEG-*b*-PLGA = 1 : 1 in mol; N_2_ pressure = 100 kPa; SPG membrane with a pore size = 1.1 μm. The drug-loading efficiency was significantly enhanced compared with the value at about 5% using traditional methods.^38,41^

As is well-known, the form of the drug is an important factor that affects the drug release profile. Thus, it is of high importance to know the form of HCPT, *i.e.* a solid solution with PEG-*b*-PLGA, an amorphous phase, or a crystalline form. Although the comet-shaped particles show birefringent properties under a polarized optical microscope, the particles are too small to achieve good quality images. X-ray diffraction was used to detect the form of HCPT within the hybrid particles (see ESI materials†). In addition to one representative peak attributed to the semicrystalline PEG-*b*-PLGA, a majority of peaks in the XRD pattern of the hybrid particles belongs to HCPT, suggesting its high crystallinity. Moreover, the HCPT within the hybrid particles shows the same polymorphic form as their bulk counterparts. In short, the XRD results nicely suggest that the growth kinetics of HCPT during the multistage crystallization are mainly changed by the active occlusion of PEG-*b*-PLGA and the confining effects of the emulsifier.

**Fig. 2:**
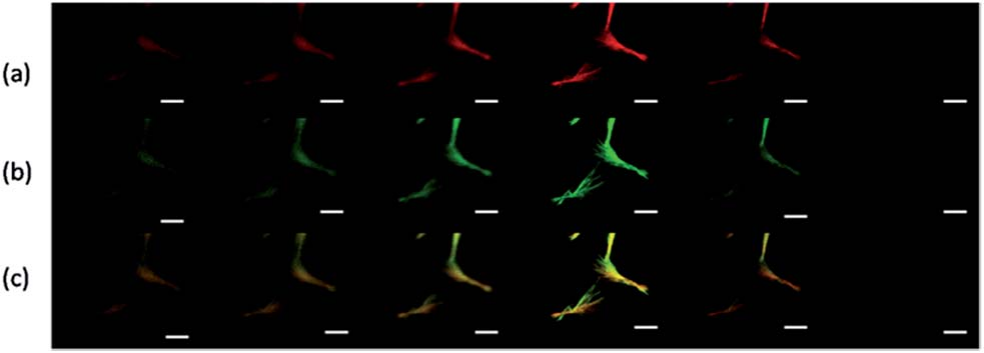
Z-scanning confocal laser scanning microscopy images of the comet-shaped particles. The images, from left to right, are taken from the top to the bottom of the particle. (a) Red fluorescence of Rhodamine B-labeled PEG-*b*-PLGA; (b) green fluorescence of HCPT; (c) the merged images of a and b. The scale bars represent 5 μm.

Next, the distribution of HCPT and PEG-*b*-PLGA within the hybrid particles has been analyzed using the Z-scanning imaging systems in Confocal laser scanning microscopy (CLSM, Olympus FV1000; Fig. 2). Green fluorescence imaging (excitation at 382 nm) was performed to visualize HCPT in the comet-shaped particles. The result nicely indicates that HCPT is uniformly distributed within the particles. To facilitate the observation of PEG-*b*-PLGA using CLSM, the polymer was labelled with Rhodamine B using the carbonyldiimidazole-mediated method (see ESI material†). Excitingly, the red fluorescence (excitation at 555 nm) overlaps nicely with the green one, presenting the homogeneous distribution of both components. The TEM image clearly indicates that the nanofibers are wrapped by a thin layer of soft ingredients, presumably PEG-*b*-PLGA (Fig. 1d). The two characterization results demonstrate the core–shell architectures of the nanofibers: the crystalline HCPT forms the core, while the shell is mainly composed of PEG-*b*-PLGA to enhance the steric stabilization of the hybrid particles in the dispersion.

**Fig. 3:**
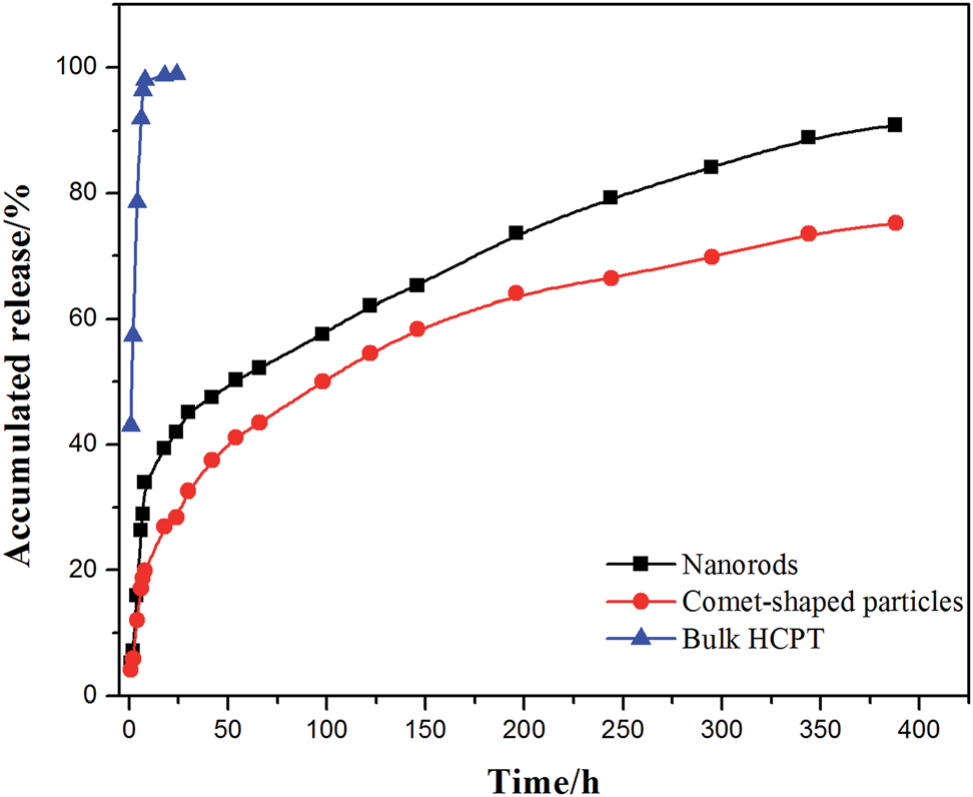
*In vitro* release profiles of comet-shaped particles, nanorods, and commercial bulk HCPT powder.

The core–shell architectures of the hybrid particles allow the sustained release of HCPT. *In vitro* release studies with the comet-shaped particles (drug loading of 16.2 wt%), alongside with the nanorods collected from the sonication-assisted precipitation (15.0% content) and bulk HCPT powder, were performed using a dialysis technique. All samples were assayed using fluorescence spectrophotometry (excitation at 382 nm). The release profiles of HCPT are shown in Fig. 3. The bulk HCPT was almost completely released within 24 h. In comparison, both the hybrid particles and the nanorods exhibit a remarkably prolonged release profile over a period of 400 h because of the limited diffusion provided by the polymeric shell. In addition, unlike the nanorods, which have a significant burst release in the initial stage, the hybrid particles exhibit a steady sustained release pattern throughout the release period. We expect that the spatial alignment of the nanofibers in the assembly effectively reduces the initial burst release compared to the isolated nanorods with a similar drug loading value, though a well-established study showed that drug release could also be controlled by the drug-loading content of the particles.^42^

The manufacturing of core–shell hybrid particles provides an increasing number of opportunities for HCPT from the practical point of view. Importantly, the anti-tumor effect of HCPT is both dose- and time-dependent.^43^ Due to its practical insolubility in the aqueous phase, people use its soluble carboxylate form for clinical treatments, usually *via* intravenous administration. Nevertheless, the carboxylate form of HCPT shows a decreased activity compared to the underivatized molecule. Other approaches show difficulties in achieving high drug-loading formulations.^38,41^ Promisingly, the sustained drug release properties and the good colloidal stability of our core–shell hybrid particles allow a local injection of the particle dispersion directly around the tumor area for sustained release at relatively low concentrations. Our cytotoxicity experiments nicely indicate that the hybrid particles show an enhanced cytotoxicity (BEL-7402) compared with the commercial HCPT (hydroxycamptothecin injection) and the colloidal PEG-*b*-PLGA (see ESI material†).

## Conclusions

The study herein presents a simple and effective approach to obtain comet-shaped drug–excipient hybrid particles with a high drug-loading content and sustained release properties. The comet-shaped HCPT–PEG-*b*-PLGA particles are promising as sustained local drug delivery system for the treatment of tumors. Animal experiments are ongoing. The integration of nanodrugs into assemblies *via* bio-inspired crystallization approaches opens a door to grow mesoscopic architectures of nanodrugs, which allow for convenient separation using current separation tools and reduced side effects compared to dispersions containing kinetically-stable isolated nanodrug particles. Co-precipitation of the drug with the excipient provides an efficient continuous manufacturing strategy to produce a new generation of drugs with high stability.
