# Supplementary material for: Integration of an anti-tumor drug into nanocrystalline assemblies for sustained drug release
Source: Chem Sci. 2015 Jan 6;6(3):1650–4. doi: 10.1039/c4sc03392b (PMC5483847; doi:10.1039/c4sc03392b)
Supplement: Supplementary file 1 [file SC-006-C4SC03392B-s001.pdf]

Electronic Supplementary Material (ESI) for Chemical Science.
This journal is © The Royal Society of Chemistry 2015

Electronic Supplementary Information

Fabrication of an Anti-Tumor Drug into Nanocrystalline Assemblies for Sustained Drug Release

Xiangrui Yang,[a] Shichao Wu,[a, b] Yang Li,[a, b] Huang Yu,[a] Di Chang,[a] Shefang Ye,[a] Liya Xie,[c] Yuan Jiang,[a] and Zhenqing Hou*[a]*

[a]Institute of Soft Matter and Biomimetics, College of Materials, Xiamen University, Xiamen 361005, China. E-mail: houzhenqing@xmu.edu.cn, yuan.jiang@xmu.edu.cn.

[b]Department of Chemistry, College of Chemistry & Chemical Engineering, Xiamen University, Xiamen 361005, China

[c]The First Affiliated Hospital of Xiamen University, Xiamen University, Xiamen 361005, China

The file includes

1. Experiemental Section

2. Tables 1-2

3. Figures 1-5

Experimental Section

Materials: All chemicals were analytical grade and used as received without further purification. The ultrapure water (18 MΩ·cm^{-1}) was used throughout the work. The 10-HCPT (purity > 99%) was purchased from Lishizhen Pharmaceutical Co., Ltd. The monomethoxy (polyethylene glycol)-poly (lactide-co-glycolide) (PEG-*b*-PLGA, PEG:10%, 5000 Da, PLGA: 28000 Da, 85/15) was obtained from Daigang Biotechnology Co., Ltd. N, N'-carbonyldiimidazole (CDI) was purchased from J&K Scientific Co., Ltd. Rhodamine B was purchased from Xinchengshi Chemical Science Co., Ltd.

Preparation of the HCPT–PEG-b-PLGA comet-shaped particles: We used the Shirasu porous glass (SPG) membrane emulsification tool to prepare comet-shaped particles. The emulsification procedure was carried out with a SPG membrane emulsification kit (SPG Technology Co., Ltd., Miyazaki, Japan). Both HCPT and PEG-*b*-PLGA were first dissolved in acetone. Afterwards, the mixture was extruded through the SPG membrane under the high N_2 pressure into the bulk water phase to grow comet-shaped particles. The final ratio of acetone/water is 1/2 in volume. After the emulsification, the remaining organic solvent was removed by the rotary vacuum evaporation at 37°C, and the resultant aqueous solution was filtered through a 1 μm cellulose acetate filter membrane to remove small drug nanocrystals as well as copolymer aggregates. The produced suspension was lyophilized for 24 h to get the dried powder. The PEG-*b*-PLGA particles were prepared with the same procedure. To obtain the favorite entrapment efficiency and drug-loading content, an orthogonal L9 (3^3) test design was used to search for the optimal formulation condition (see details below). As shown in Table S1, three parameters including the mass ratio of HCPT to PEG-*b*-PLGA, the N_2 pressure, and the pore size of the SPG membrane have

been tested in the preliminary experiments to find out the favorite experimental conditions.

Preparation of the HCPT–PEG-b-PLGA nanorods: The HCPT–PEG-*b*-PLGA hybrid nanorods were prepared via an ultrasound-assisted emulsion crystallization technique followed by the lyophilization treatment. Briefly, PEG-*b*-PLGA and HCPT were dissolved in 20 mL of acetone, which was then added dropwise into deionized water 50 mL in volume in 5 min with sonication at the power of 200 W in an ice bath. Afterwards, the mixture was stirred overnight at RT to allow acetone to evaporate. After further evaporation of acetone in a vacuum oven, the obtained dispersion was frozen at -80°C and lyophilized for 24 h to obtain the dried powders.

Synthesis of the PEG-b-PLGA–Rhodamine B: CDI (80 mg, 0.25 mmol) was dissolved in dimethylformamide (DMF, 10 mL) in the N_2 atmosphere as the stock solution A. PEG-*b*-PLGA (1000 mg, 0.03 mmol) was dissolved in DMF (10 mL) as the stock solution B. Afterwards, the two stock solutions were mixed with stirring in the N_2 atmosphere for 4 h at RT. The remaining CDI was removed by dialysis in deionized water at 4°C for 12 h, and the dialysate was changed every hour. The suspension was centrifuged at 20,000 rpm (Beckman) at 4°C for 30 min and washed thoroughly with the distilled water for three times. The remaining product was frozen in the liquid N_2 and lyophilized for 24 h.

A mixture of the excessive Rhodamine B and activated PEG-*b*-PLGA was codissolved in acetone and stirred under nitrogen at RT for 48 h. The unreacted Rhodamine B was removed by dialysis in deionized water at 4°C for 12 h, and the dialysate was changed every hour. The suspension was centrifuged at 20,000 rpm (Beckman) at 4°C for 30 min and washed thoroughly with distilled water for three times. The product was frozen in liquid N_2 and lyophilized for 24 h.

Characterization: The morphology of the HCPT–PEG-*b*-PLGA particles was examined by SEM

(LEO1530) and TEM (JEM-2100) at 20 and 200 kV, respectively. In the fluorescence optical microscopy measurements, the fluorescence detection wavelengths of HCPT were set at 382 nm (excitation; emission: 528 nm), and the fluorescence detection wavelengths of HCPT were set at 555 nm (excitation; emission: 580 nm). The fluorescence distribution of the HCPT–PEG-b-PLGA particles was obtained by scanning in the Z-direction with the step length at 100 nm from the top surface to the bottom. All Z-section images were captured under the same resolution and at the same position. The average particle size and size distribution of the comet-shaped particles were determined by photon correlation spectroscopy with a Malvern Zetasizer Nano-ZS (Malvern Instruments, Malvern) at 25°C under suitable dilution conditions. Measurements were repeated for three times to get the consistent results.

The amount of HCPT entrapped in the particles was determined indirectly by UV spectrophotometry (Beckman DU800). All samples were assayed at 383 nm. The weight of the drug entrapped in the particles was calculated by the calibration curve. Drug-loading content and entrapment efficiency were presented by Equations (1) and (2):

$$Drug\ loading\ content\ (\%) = (weight\ of\ drug\ in\ NPs)/(weight\ of\ NPs) \times 100\% \quad (1)$$

$$Entrapment\ efficiency(\%) = (weight\ of\ drug\ in\ NPs)/(weight\ of\ feeding\ drug) \times 100\% \quad (2)$$

The crystalline form of HCPT loaded in HCPT–PEG-b-PLGA particles was analyzed using XRD (X'pert PRO). The X-ray diffractogram was scanned with Cu-ka radiation generated at 30 mA and 40 kV. The diffraction angle was increasing from 5 to 60 degree, with a step size of 0.016 degree.

In Vitro Drug Release Studies: The *in vitro* drug release studies of particles were performed using the dialysis technique. The comet-shaped particles were dispersed in PBS (10 mL) and placed into a pre-swelled dialysis bag (MWCO 3500 Da). The dialysis bag was then immersed in 0.1 M PBS at pH 7.4,

and oscillated continuously in a shaker incubator (100 rpm) at 37°C. All samples were assayed by fluorescence spectrophotometry. The nanorods containing the equivalent concentrations of HCPT were used for comparison.

Cytotoxicity of HCPT–PEG-b-PLGA Comet-shaped particles: The cytotoxicity of comet-shaped particles was determined by the MTT assay. Briefly, an adequate number of exponential phase cells was plated in quintuplicate in a 96-well flat bottomed microplate and incubated for 24 h in the culture solution containing comet-shaped particles, nanorods or bulk HCPT. In this study, 20 mL of MTT (Sigma) solution (5 mg/mL in PBS) was added in each well, and the plates were incubated at 37°C for another 4 h. This was followed by the addition of 150 mL dimethylsulfoxide (DMSO) and the plate was agitated on a water bath chader at 37°C for another 30 min. The absorbance at 570 nm was measured using a Microplate Reader (model 680; Bio-Rad).

2. SUPPORTING TABLES

Reaction conditions in Figure 1

The samples of image a-c were prepared under the experimental conditions as follows: HCPT / PEG-b-PLGA = 1 / 1 in mole; nitrogen pressure = 200 kPa; SPG membrane with the pore size = 1.1 μm. The samples of image d-e were prepared under the experimental conditions as follows: HCPT / PEG-b-PLGA = 1 / 1 in mole; nitrogen pressure = 100 kPa; SPG membrane with the pore size = 1.1 μm.

Table 1. Factor-lever in orthogonal-design experiments L9 (3^3)

level	Factor		
	A<br>HCPT/PEG-*b*-PLGA(*w/w*)	B<br>N_2 pressure (kPa)	C<br>pore size (μm)
1	1:2	50	0.4
2	1:1	100	0.6
3	2:1	200	1.1

Table 2. Result of orthogonal-design experiments L9 (33)

No.	A	B	C	Drug-loading (%)	Entrapment efficiency (%)
1	1(1:2)	1(50)	1(0.4)	13.1±1.2	39.3±3.6
2	1(1:2)	2(100)	2(0.6)	31.7±0.7	95.1±2.1
3	1(1:2)	3(200)	3(1.1)	32.2±1.8	96.6±5.4
4	2(1:1)	1(50)	3(1.1)	22.4±2.3	44.8±4.6
5	2(1:1)	2(100)	1(0.4)	46.7±2.7	93.4±5.4
6	2(1:1)	3(200)	2(0.6)	47.3±1.9	94.6±3.8
7	3(2:1)	1(50)	2(0.6)	25.8±2.4	38.7±3.6
8	3(2:1)	2(100)	3(1.1)	59.2±3.8	88.8±5.7
9	3(2:1)	3(200)	1(0.4)	51.9±3.2	77.9±4.8

3. SUPPORTING FIGURES

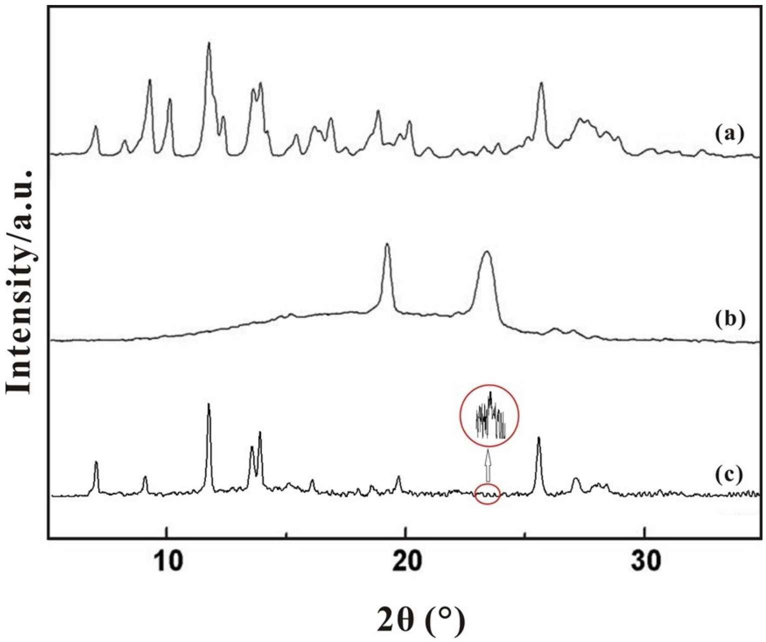

Figure 1. The XRD patterns of a) bulk HCPT. b) PEG-*b*-PLGA. c) comet-shaped particles.

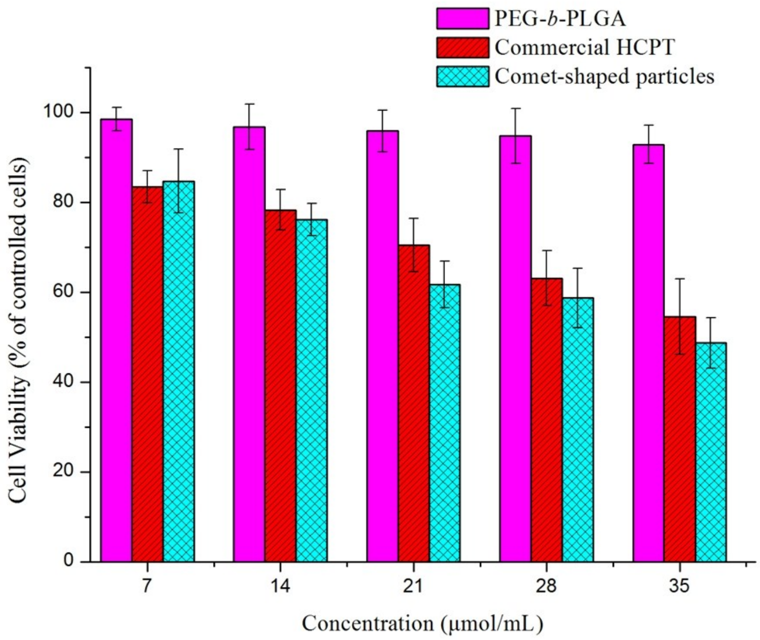

Figure 2. The *in vitro* cytotoxicity assay against human liver BEL-7402 cells (24 h). $P < 0.01$. The drug-loaded content of comet-shaped particles is 16.2%.

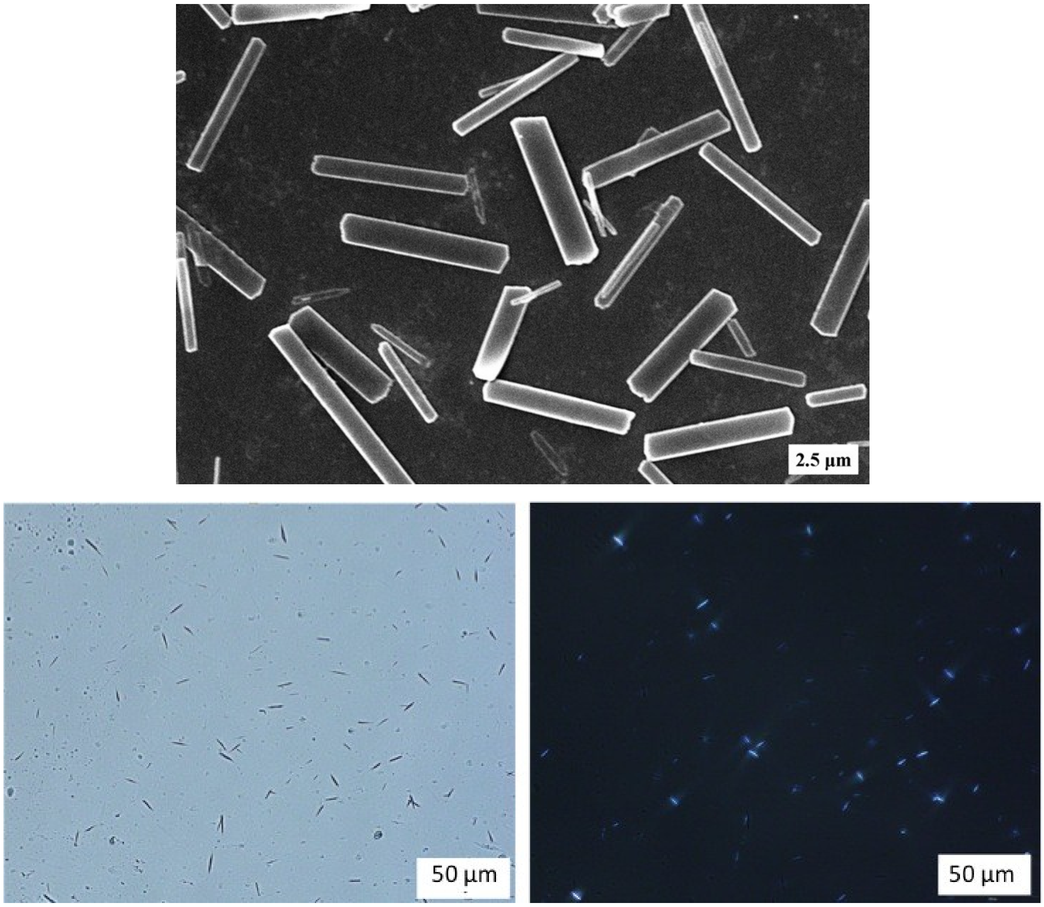

Figure 3. SEM (above), OM, and POM (bottom) images of HCPT–PEG-*b*-PLGA hybrid nanorods prepared via a sonication-assisted precipitation process. Both OM and POM images were taken at the same position by using the freshly prepared sample.

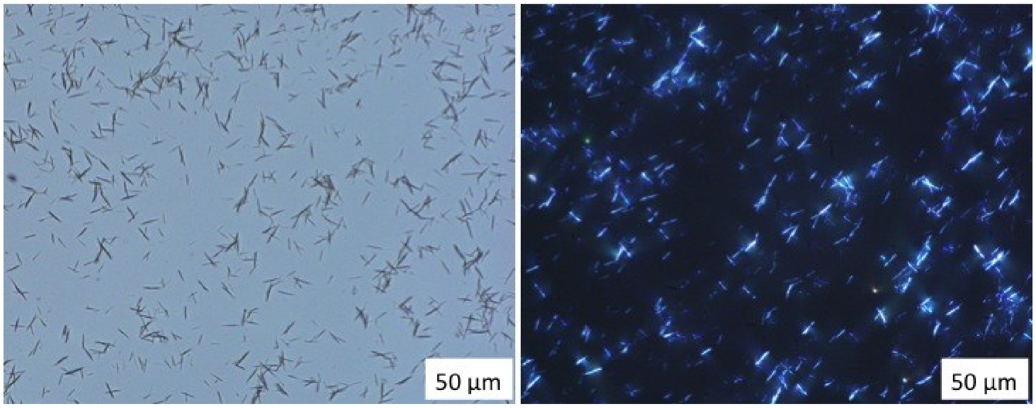

Figure 4. The OM and POM images of HCPT–PEG-*b*-PLGA hybrid nanorods prepared via an anti-solvent precipitation process under the stagnant conditions. Both OM and POM images were taken at the same position.

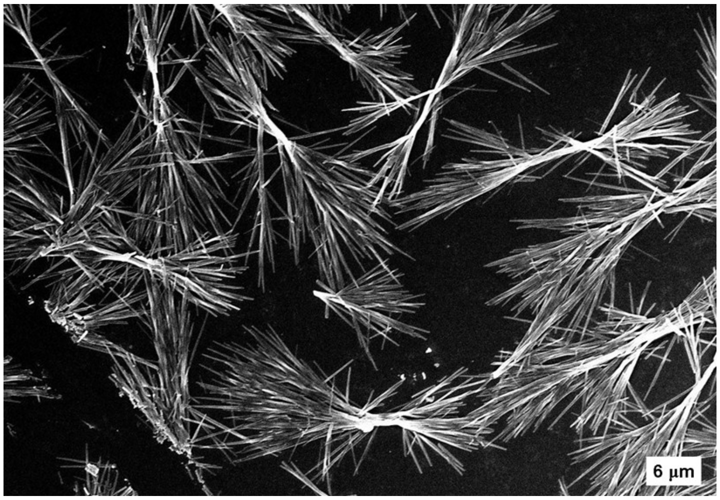

Figure 5. The SEM image of the dumbbell-shaped particles achieved after pumping the freshly prepared hybrid nanorods acetone-water dispersion through a 1 μm filter membrane into the saturated HCPT acetone-water mixture.
